# eHealth in Latin America and the Caribbean: Development and Policy Issues

**DOI:** 10.2196/jmir.5.1.e4

**Published:** 2003-03-31

**Authors:** Roberto J Rodrigues, Ahmad Risk

**Affiliations:** ^1^Pan American Health Organization / World Health OrganizationDivision of Health Systems and Services DevelopmentWashington DCUSA; ^2^World Health OrganizationDepartment of Health Information Management and DisseminationGeneva 27Switzerland

## Abstract

This paper reviews trends and issues in health and in the information and communication technologies (ICT) market as they relate to the deployment of eHealth solutions in Latin America and the Caribbean. Heretofore designed for industrialized countries and large organizations, eHealth solutions are being proposed as an answer to a variety of health-system management problems and health care demands faced by all health organizations including those in developing societies. Particularly, eHealth is seen as especially useful in the operational support of the new health care models being implemented in many countries. The authors examine those developments vis-à-vis the characteristics of the Latin American and the Caribbean health-sector organizational preparedness and technological infrastructure, and propose policy and organizational actions to foster the development of eHealth solutions in the region.

## The Foundation and Practice of eHealth

Advances in ICT and the dissemination of networked data processing created a new environment of universal access to information and the globalization of communications, businesses, and services. In the health sector, this trend is exemplified by the growing consolidation of eHealth - an area distinguished by the combined utilization of electronic communication and information technology to transmit, store, and retrieve digital data for clinical, educational, and administrative purposes, both at the local site and at distant sites. Barely in use before 1999, the concept of eHealth evolved to serve as a general designation to characterize not only networked health applications, but also virtually everything related to computers and medicine. It followed a trend started by other "e-words," such as e-Commerce, e-Business, e-Finance, e-Learning, e-Government, e-Solutions, and e-Strategies [1- 3]. Among leading digital technologies, Internet-based ICT solutions are rapidly changing the way health providers, health plans, organizations, payers, regulators, and consumers access information, acquire health products and services, deliver care, and communicate with each other [4,5].

Most eHealth solutions build on e-Commerce and e-Government strategies and experiences in using Internet-based networked technologies to rethink, redesign, and rework how businesses and public services operate. Typically, such developments have been aimed at the improvement of productivity, effectiveness, and efficiency, both internally and in the relationships with clients, customers, suppliers, and partners. Reaping the full benefits of such innovative data processing and use depends on the clear definition of goals, collaboration among stakeholders, technology infrastructure, systems integration, standards, and the implementation of performance metrics.

The business imperative for eHealth is concrete, is driven by the operational requirements of health reforms, and is aligned to many of the determinants found to be relevant in e-Commerce [6- 8]:

- Growth of a global marketplace and the ubiquity of interactive communications.
- Networks of producers, suppliers, customers, and clients.
- Global demand for telehealth services is estimated to be of US $1.25 trillion, of which about two-thirds is for direct services and the rest for second opinion, consumer information, continuing education, management, and other services.
- Leasing, membership, service agreement, and strategic-alliance models replace traditional business organizations based on ownership of physical assets and long-term structures.
- Lifetime value of customer retention replaces "one-time sell."
- Economies of speed, forecasting demand, and customer service and satisfaction replace economies of scale and impersonal service provision.
- Customization capable of achieving a "one-of-a-kind" product or service.
- Leveling effect by reducing entry barriers, thus allowing small firms and poor countries and populations to have access to markets, information, and other resources, and therefore balancing the vertical integration competitive advantage of large corporations.

The essence of eHealth, as in e-Commerce, is reliable transaction delivery in a fast-changing environment involving people, processes, and a business infrastructure focused on the ill or healthy citizen. In developed countries, eHealth has rapidly evolved from the delivery of online medical content toward the adaptation of generic e-Commerce solutions to the processing of health-related administrative transactions and logistical support of clinical tasks. Emerging eHealth applications are oriented to professional networking, integration of the clinical care process management, and the provision of Web-based health information and patient care, including remote monitoring and health care. This expanded view of eHealth has been promoted as the final stage in bringing the entire health care industry online.

## Organization and Delivery of Health Care in Latin America and the Caribbean

The health sector faces two demands that appear, on first examination, to be contradictory: firstly, to provide expanded and equitable access to quality health care services and, secondly, to reduce or at least control the rising costs of health care services. Although the health sector is key to the welfare of the population and the formation of human capital, the sector has not kept pace with the momentum of change that the region has experienced in recent years in other areas of economic, political, and social life.

Health sector expenditures in Latin America and the Caribbean comprise 6% to 17% of the service sector that, in turn accounts for 50% to 65% of the GDP (Gross Domestic Product) in almost all countries. The market for health goods and services in the countries of the region represent 9% of the global health market, about half of that for the European Union, above that of the combined markets of Eastern Europe and Central Asia, and just below that of East Asia and the Pacific. The average per capita expenditure on health in 1999 was US $452 followed by US $1868 for the European Union, US $2206 for Canada, and US $3978 for the United States. (PPP dollars). PPP (Purchase Power Parity) relates to the purchase value of the dollar at the local market. PPP rates allow a standard comparison of real price levels between countries. The PPP for a specific country is based on market price surveys conducted by the International Comparison Program, a joint project of the World Bank and the regional economic commissions of the United Nations. The present values of PPP are based on the last survey done in 1996 and is based on a 1993 reference year.

**Figure 1:**
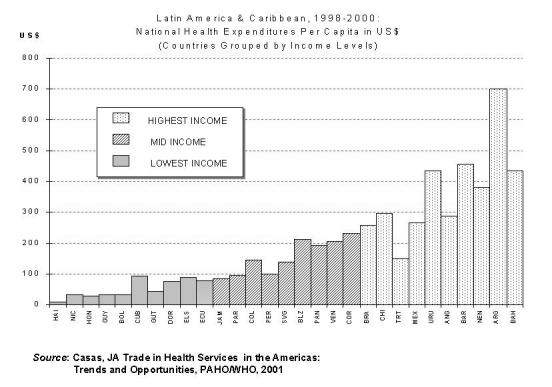
Even among countries of similar income level there is a marked variation in the per capita health expenditure. From Casas JA. Trade in health services (THS) in the Americas: trends and opportunities. Paper presented at International Summit on the Private Health Sector; 2001 Dec 2-5; Miami, Fla

There is a marked variation (Figure 1) in the national expenditures among Latin American and the Caribbean countries even for countries of comparable income level [9].

Changing demographics (particularly age structures) and lifestyles (mainly due to epidemiological profiles, urbanization, and growing industrialization) highlight the need to reorient care models. In high-income and middle-income countries, about 40% of the population has one or more chronic conditions and, in many societies, chronic conditions account for up to two-thirds of health care expenditures. In each care setting, a limited set of health conditions account for most of the cost due to the growing demand on the care system for additional and high-cost diagnostic and therapeutic resources. Significant opportunities do exist to improve health status but there are still a considerable number of preventable diseases and premature deaths, both in absolute and relative terms, and there is great inequity of access to basic health services resulting in regions, communities, and social groups being left without access to the most basic health care.

In most countries, the health sector is underfinanced; this has led to quantitative and qualitative deficiencies in the delivery of health services and to growing gaps in basic care. There is inefficient allocation of scarce resources and lack of coordination between health subsectors, institutions, and other social agents and stakeholders - with duplication of efforts, overlapping responsibilities, and resource wastage. All countries are in some stage of sector reform, a process aimed at introducing substantive changes in the health sector and in the relationships among stakeholders and the roles they perform, with a view to increasing equity in benefits, efficiency in management, and effectiveness in satisfying the health needs and expectations of the population [10].

Health reform processes have many facets and there is no single model being adopted by all countries. Each country is moving at a different pace in the implementation of its own particular health system model but the economic and globalization changes of the last 10 years have brought a new urgency to the reform processes. There are, however, common trends and responses that characterize most health sector reform processes occurring in the region: (a) the universalization of a high cost-benefit basic package of health services; (b) a set of standardized public health interventions; (c) cost containment and recovery; (d) administrative decentralization and operation of health care services; (e) recognition of the role of the private subsector and the intersectorality of health interventions (involvement of other sectors - for example, education, environment, and labor - besides health in the determinants of health status and health care activities); (f) health models oriented towards primary care and centered on people; (g) focus on quality and accountability; and (h) moving away from the reactive delivery of care to a more proactive management approach of the health status of individuals and population groups [10,11]. With the introduction of ICT, health sector users (clients) and providers (professionals and organizations) will be forced to assume new roles (different from the traditional ones). Furthermore, the introduction of ICT will bring into the health sector environment a host of new professionals (eg, systems professionals) and will require novel forms of interaction with municipal and provincial (state) health authorities. Competition, merger of provider organizations, aggressive contracting by payers, and increasing involvement of employer and government purchasers have characterized changing processes in health services management. Information systems are essential for operational support and management of the new health and health care models [11- 15]. They must address the needs of the new trends in health care that emphasize a continuous relationship between providers and clients; customization of care; expanding partnering of providers, insurers, and clients; increasing client control of evidence-based health decisions; information that is not frozen in records and kept in separate sites with access limited to their creators but available to all stakeholders; and transparency and cooperation instead of independent professional roles.

## Challenges to the Deployment of eHealth in Latin America and the Caribbean

### Socioeconomic and Development Constraints

**Figure 2:**
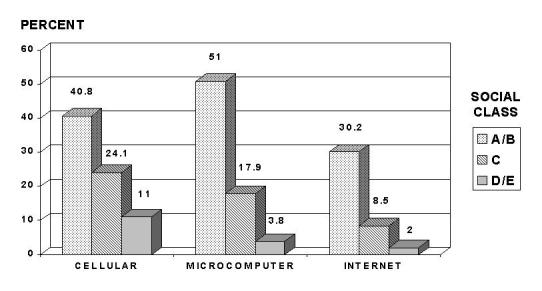
Use of digital technologies by social class in Brazil. Categorization of social classes according to the classification adopted by the Brazilian Institute of Geography and Statistics (IBGE), similar to the quintile categorization for incomes where A corresponds to the upper quintile, and E for the lowest quintile of household income. From Ministério da Saúde (1999). Cartão Nacional de Saúde, Concorrência Internacional 001/99, Anexo: Doicumentação, Brasília DF [Ministry of Health of Brazil]

Technology distribution and access problems represent the most acute issue in the dissemination of ICT applications. In a more limited focus, the "digital divide" encapsulates the dramatic worldwide variation in access to computer-based information technologies, usually more narrowly in terms of levels of Internet access, available to individuals and communities. Information-technology utilization inequalities are found in both industrialized and developing countries and are determined by level of education and income (Figure 2).

Digital divides, like social and economic divides, exist within and not just between societies and are integral parts of a much broader and intractable "development divide" that include insufficient telecommunications infrastructure, high telecommunications tariffs, inappropriate or weak policies, organizational inefficiency, lack of locally-created content, and uneven ability to derive economic and social benefits from information-intensive activities [16- 18]. The situation of technology adoption within developing countries has been one of growing polarization, with segments of the population bypassed by the products of the information revolution. This is complicated by the fast-changing deployment of new technologies and accompanying standards that are constantly raising the level of advancement that must be met by anyone who wants to remain current [19,20].

### Technology Infrastructure, Market Determinants, and Operational Issues

In the health sector, development and digital divides between industrialized and Latin American countries are wider than the gap observed in other productive and social sectors. In some cases, the changes brought about by the privatization of health care did add to the already high degree of structural inequity that prevails in the countries of the region. Besides achieving reliable transaction delivery, a technologically successful "e-Architecture" must provide superior client service, customization of products and services, interactivity, and maximum convenience [21].

**Table 1:** Legal ownership of 16566 hospitals and computerize dinformation systems in Latin America and the Caribbean; 1995 to 1997*

**Ownership Class**	**Hospital Groups**							
	**All Facilities**		**No Computers**			**With Computers**		
	Number	Percent	Number	Percent		Number	Percent	
				In Class	In Group		In Class	In Group
Public non-social security †	6498	39.22	5099	78.47	44.98	1399	21.52	26.74
Public social security	876	5.29	438	50.00	3.86	438	50.00	8.37
Private	7783	46.98	4924	63.26	43.43	2859	36.73	54.66
Philanthropic	1284	7.75	779	60.66	6.87	505	39.33	9.65
Military	125	0.75	96	76.80	0.84	29	23.20	0.55
Total	16566	100.0	11336		100.00	5230		100.00

^*^ From PAHO Directory of Latin American and Caribbean Hospitals Database.

^†^ Social security = health care subsector supported by contributions from employees and employers through a national insurance scheme.

**Table 2:** Hospital size in Latin America and the Caribbean by number of beds; 1996-1997*

**Number Of Beds**	**Hospitals**		**Beds Available**	
	**Number**	**Percent**	**Number**	**Percent**
1-50	10027	60.5	219383	20.0
51-100	2615	15.8	189559	17.3
101-200	1703	10.3	242770	22.1
201-300	544	3.3	133225	12.1
301-400	242	1.5	84811	7.7
401-500	133	0.8	58951	5.4
501-1000	186	1.1	126169	11.5
>1000	29	0.2	43097	3.9
Subtotal	15479	93.4		
No data	1087	6.5		
Total	16566	100.0	1097965	100.0

^*^ From PAHO Directory of Latin American and Caribbean Hospitals Database.

The deployment and operation of "e-Solutions" share technology-infrastructure and operational-deployment issues involving reliability of service that directly depend on: (a) degree of information preparedness and information technology insertion in society; (b) appropriate and functioning network, hardware and software platforms, and physical infrastructure; (c) the understanding of market relationships among the different actors in the informatics and telecommunications areas; (d) managing knowledge about health, individual client medical history, environment, and enterprises; (e) data-protection measures and regulatory framework to ensure transaction security; and (f) auditing processes that are quite different from traditional paper-trail solutions. A pervasive public-sector information infrastructure ("infostructure"), the essential prerequisite for continuous health care to the community, is still an incipient component of health systems. Penetration of information systems in health institutions is low. The hospital subsector is the area better served by information systems. Table 1 summarizes the distribution of computerized systems in hospitals.

Considering all facilities, public hospitals, including those belonging to the social security, account for 44.51%; private total 46.98%; philanthropic total 7.75%; and military the remaining 0.75%. There are significant differences in the utilization of computerized information systems. The relative distribution of information systems shows that social-security hospital facilities constitute only 5.29% of all establishments, but 50% have computerized information systems, followed by philanthropic (39.3%), private (36.7%), military (23.2%), and public non-social security (21.5%). However, in the group of all hospitals with computerized systems, private hospitals represent the majority (54.7%). The disparity between the existence of systems in the two types of public hospitals (public social security and public non-social security) is evident.

Facility size is a major determinant in the capability of an institution to implement ICT and in the selection of the portfolio of applications. Of all Latin American and Caribbean hospitals, 10027 (60.53%) have 50 or fewer beds (Table 2) and of those, 5621 (56%) are private, 3806 (37.95%) are public, 529 (5.27%) are philanthropic, and 71 (0.7%) are military.

**Table 3:** Expenditures on information and communication technologies in selected countries*

Country	ICT Expenditures Per Capita in US$ (2000)	ICT Expenditures as % of GDP (2000)
Argentina	317	4.1
Brazil	289	8.4
Chile	360	7.8
Colombia	228	12
Mexico	189	3.2
Venezuela	196	3.9
Australia	1922	9.7
Austria	1697	7.2
Belgium	1769	8
Canada	1911	8.4
China	46	5.4
Finland	1835	7.8
France	1916	8.7
Germany	1798	7.9
Hungary	431	8.7
Italy	1068	5.7
Japan	3118	8.3
Norway	2445	6.9
Russian Fed	63	3.7
Singapore	2104	9.7
Spain	731	5.1
Sweden	2674	10.4
United Kingdom	2187	9.1
United States	2296	8.1

^*^ From World Bank. World development report 2000/2001 - attacking poverty, Oxford University Press; 2001.

**Table 4:** Technological preparedness in selected countries of Latin America and the Caribbean*

Country	Number of Fixed Telephone Lines x 100 persons(2001)	Change in Number of Fixed Telephone Lines x 100 persons (1995-2001)	Faults per 100 Fixed Lines per Year (2000)	Percentage Households with Telephone (2000)	Subscription as % of Per Capita Income (1999)	Telecom Investment Per Capita in US$ (2000)	Telecom Investment as % of Revenue (2000)	Numbers of Personal Computers Per 100 Persons (2001)	Internet Users x 1,000 (2001)	Internet Users Per 100 Persons (2001)
Antigua & Barbuda	47.35	3.4	59		1.5				5	6.52
Argentina	21.63	5	17.3		2.1	51.4	19	5.34	3000	8
Bahamas	40.03	4.9							16.9	5.49
Barbados	46.29	6			2	100.6	15.8	9.33	10	3.74
Belize	14.44	1.3	65.6	53	1.6	31.5	15.1	13.52	18	7.38
Bermuda	87.15	2.8				443.6	33	49.54	25	39.01
Bolivia	6.04	10.4			2	13.8	30.7	2	120	1.44
Brazil	21.69	16.9	2.8	37.6	2.3	52	34.8	6.26	8000	4.64
Chile	23.9	11.1	25	52.6	2.9	71.9	43.1	8.39	3102.2	20.02
Colombia	17.05	9.2	59.9		2	27.8	40.7	4.21	1154	2.7
Costa Rica	22.97	8.1	65.1	54.3	3.8	34.7	58	17.02	384	9.34
Cuba	5.1	8	10	10.1	5.3	9.6	15.3	1.96	120	1.07
Dominica	29.06	3.1						7.5	6	7.78
Dominican Rep.	10.84	6.4							186	2.15
Ecuador	10.37	9.3	48	8.9	1.8	3.6	9.8	2.33	327.7	2.54
El Salvador	9.34	10.9	14.5		4.3	139.5	156	2.19	50	0.8
Grenada	32.75	3.9	9	90	4.6			13	5.2	5.2
Guadeloupe	44.93	2.9				48.2	15.7	21.74	8	1.75
Guatemala	6.47	14.5			-			1.28	200	1.71
Guyana	9.19	9.4	87		0.3	78.1	70.2	2.64	95	10.92
Haiti	0.97	2.5							30	0.36
Honduras	4.71	9.7	24	16.4	3	16.8	54.5	1.22	40	0.62
Jamaica	19.73	9.2	48			53	29.4	5	100	3.85
Mexico	13.48	6.2	1.9	36.2	3.5	51.4	38.7	6.87	3500	3.49
Nicaragua	3.12	7.1	79.3		19.6	0.3		0.96	50	0.99
Panama	14.83	4.2	48		1.1			3.79	90	3.17
Paraguay	5.12	6.8		18.4	4	15.4	24.9	1.42	60	1.06
Peru	7.75	8.6	17.1	23.8	7.4	17	31.1	4.79	3000	11.5
St. Kitts and Nevis	56.88	9.4				0.2	-	19.05	2	5.16
St. Vincent & Grenadines	21.96	5.9	8.6	90	3.1	38.6	15.2	11.61	3.5	3.09
Suriname	17.58	4.9	30.9		0.5	43.3	40.9		14.5	3.3
Trinidad & Tobago	23.99	6.1	75		1.2	73.7	39.5	6.92	120	9.23
Uruguay	28.29	6.4	5.6	75	1.6	31.4	13.5	11.01	400	11.9
Venezuela	11.2	-0.3	2		2.6	12.1	8.1	5.28	1300	5.28
WORLD	17.19	5.8	24.8		5.7	35.7	22.1	7.74	498665.7	8.21
Africa	2.62	6.7	35.7		12.7	6.2	25.2	1.06	6867.7	0.85
Americas	35.14	3.4	13.8		3.1	68.3	14.2	26.57	182514.1	21.64
Asia	10.68	12	60.3		5.5	25.1	38.4	2.18	156508.5	4.34
Europe	40.54	3.3	19.8		1.1	66.6	19.2	17.94	144413.1	18.05
Oceania	40.04	0.5	5.9		3.7	137.7	23.5	39.91	8504.3	27.72

^*^ From World Bank. World development report 2000/2001 - attacking poverty. Oxford University Press; 2001. And from World Telecommunication Indicator Database [CD-ROM]. 6th ed. Geneva: International Telecommunication Union; 2002.

In the past decade, the information technology sector in Latin America and the Caribbean consistently showed a growth of 17%, above all other world regions. During the last 5 years there has been fast expansion of the telecommunications market (from 7 million fixed-line and 50 million mobile subscribers in 1995 to 25.3 million and 69 million subscribers respectively in the year 2000) and very fast diffusion of the Internet (in 3 countries alone - Brazil, Mexico, and Argentina - the total number of registered geographic domain hosts grew from 326000 in July 1998 to 660000 in July 1999). Investments in ICT as percentage of the GDP is comparable to that of developed countries although the absolute value per capita is low (Table 3). Data used in the preparation of Table 3 come from sources that in one case use GNP (Gross National Product) and in the other case use GDP. GNP is the broadest measure of national income and measures total value added from domestic and foreign sources claimed by residents. GNP comprises GDP plus net receipts or primary income from nonresident sources

Low penetration of telephony, averaging 12% in the region and cost of annual subscription (averaging 3% to 4% of the GNP per capita, but in some countries as high as 19.6%), and very low ownership of personal computers (2 to 10 for each 100 persons, average 3%), and low Internet connectivity (average 3%) are major challenges to be overcome (Table 4). About 2 out of 3 public ICT projects are deemed to be failures - they take a long time to implement, cost more than planned, and deliver less than planned. Most of the problems are related to the process of bidding, selection, and contracting.

Poor telecommunications infrastructure, limited number of Internet Service Providers (ISPs), lack of access to international bandwidth, and lack of affordable Internet-access costs are readiness issues that continue to be major impediments to diffusion of Internet applications to the point of care in developing societies. Dependable connectivity is needed for reliable transactions. Fast connectivity is still limited and is usually by dial-up access. A study across different industries showed that only about one-third of the connected organizations in the region had access with speed higher than 56 Kbps (Kilobits per second) (Table 5).

**Table 5:** Connectivity speed in selected countries

Country	>Organizations with Access > 56 Kbps*
Mexico	42%
Peru	39%
Chile	37%
Brazil	33%
Argentina	31%
Colombia	31%
Venezuela	27%
Ecuador	22%
Regional average	35%
* Kilobits per second	

^*^ From Harte-Hanks 2001 CI Technology Database. Harte-Hanks Market Intelligence, a fee-based Web service at URL: http://accessCI.hartehanksmi.com/

The access-site problem can be further illustrated by the result of a 1999 survey of 42744 physicians in Brazil (done by a pharmaceutical company in Brazil). The study revealed that 52% used the Internet - a level of diffusion equivalent to the general US population - however, when 23603 physicians users were asked from where they predominantly accessed the Internet, 85% indicated their home, 10% the office, and only 2% and 3% indicated the site as the university or the hospital respectively. In comparison, US physicians have the following Web access profile: 40% at the workplace, 56% at the office, 87% at home, and only 7% were not connected.

On a positive note, reform of the telecommunications sector is bringing significant improvement in services and drop in tariffs as a result of greater competition and expanding markets. With the recent rapid trade liberalization and modernization of the telecommunications sector in Latin America and the Caribbean, the telecommunications infrastructure is improving. One-fourth of the 89 major public telephone operators that were privatized throughout the world by the end of 1999 were in Latin America and the Caribbean [17].

**Table 6:** Technology exports, royalties, and licenses payments for the year 2000*

Countries	High Technology Exports at % of All Manufactured Products Exported	Royalties and Licenses Income in Millions of US$	Royalties and Licenses Payments in Millions of US$	Royalties and Licenses Balance in Millions of US$	Gross National Income in Billions US$	Royalties and Licenses Balance as % of GNI
Argentina	9	13	458	-445	276.2	-0.16
Bolivia		2	5	-3	8.2	-0.04
Brazil	19	126	1415	-1289	610.1	-0.21
Chile	3	102	44	58	69.8	0.08
Columbia	7	4	71	-67	85.3	-0.08
Costa Rica		1	31	-30	14.5	-0.21
Dominican Republic			30	-30	17.8	-0.17
Ecuador	6		62	-62	15.3	-0.41
El Salvador	6	2	20	-19	12.6	-0.14
Honduras	2	0	10	-10	5.5	-0.18
Jamaica	0	6	41	-35	6.9	-0.51
Mexico	22	43	407	-364	497	-0.07
Panama	0	0	30	-30	9.3	-0.32
Peru	3		57	-57	53.4	-0.11
Uruguay	2		11	-11	20	-0.06
World						
Low and Middle Income	16	1873	11064	-9191		
East Asia & Pacific	25	784	5409	-4625		
European and Central Asia	10	313	1753	-1440		
Latin America & Carribean	16	501	2666	-2186		
Middle East & Northern Africa	1	106	614	-508		
South Asia	3	87	338	-251		
Sub-Saharan Africa	8	82	283	-201		
High Income	22	70321	62988	7333		
European Commmunity	16	11019	23422	-12403		
United States	34	33030	16100	21930		
Japan	28	10227	11007	-730		

^*^ From World Bank. World development report 2000/2001 - attacking poverty, Oxford University Press; 2001.

### Impact in the Society and in Health Practice

Many market segments are becoming increasingly information-technology dependent as part of globalization [7,22- 24]. Areas of concern in the introduction of electronic marketplaces, particularly in developing countries, are related to the difficulties in regulating offshore business, the dominance of the Internet global-communications infrastructure by a few countries, and growing concentration of power and knowledge in few corporations.

As is usually the case with innovation, the agents that first move into the market quickly attain a dominant position, block the entry of new competitors, and capture a large part of potential proceeds. It has been stated that the success of developed countries, particularly the United States, in taking advantage of ICT partly reflects its flexible and competitive markets. Possibly, smaller benefits can be expected in more-regulated economies or in the case of implementation environments characterized by rigid labor and trade rules and by inefficient commodity markets and capital exchanges [25].

Cross-border challenges are particularly pressing due to the growing number of national, international, and nongovernmental actors involved in transnational and global concerns. Market capture by strong, organized, and well-funded health-provider organizations, some of international nature, is happening at a fast pace in Latin America and regulatory methods have been advocated to safeguard local competition. Intangible health "e-Solutions" products and services offered by foreign providers - as is the case with investment, insurance, knowledge dissemination, and health care applications - present great challenges to developing and poorly-developed countries and may result in flight of capital, tax evasion, employment reduction, capture of the health market, and "cultural colonization."

In the area of information technology, the emphasis of intellectual rights has changed from the protection of the author/inventor to that of the investor. Implications for developing countries [26], welfare effects, foreign direct investments, transfer of technology, and impact on domestic markets are difficult to foresee particularly in relation to foreign direct investments and technology transfer. Even countries with significant technology exports show negative net balance of royalties and license fees (Table 6).

Intellectual property rights have been a major area of concern and conflict. The promotion of innovation is essential to any development strategy, particularly to increase international competitiveness of national enterprises. However, limiting acquisition of innovative technology only to those that are captured by the patent system ("inventions") makes a society permanently dependent on external sources. The universalization of standards for protection of intellectual-property rights has been enabled by the World Trade Organization General Agreement on Trade and Services (GATS) adopted in 1995 at the Uruguay Round of negotiations, reinforcing protection in 3 key information technology areas: computer programs, databases, and design of the layout of integrated circuits. Stakeholders concerned with such issues include nation-states; multinational business organizations; subregional trade blocks, and integration groups (formal trade blocks such as NAFTA [North American Free Trade Agreement], Mercosur, and the European Union Common Market, as well as other regional integration initiatives such as Andean Countries and CARICOM [Caribbean Common Market]). In addition, country initiatives, professional and nongovernmental groups, and international organizations provide an operational and legal framework for tackling these issues. Some of these entities are being increasingly overwhelmed by market access challenges not envisioned before the diffusion of ICT.

### Skilled and Committed Human Resources are Essential

People are central in the value-added creation of eHealth products and services and an organization's human resource is the key to success [3]. Systems professionals, technology products and services providers, and project teams must also have superior skill levels and experience in the particularities of the area being automated. Regarding the number of technicians, scientists, and portion of the GNP devoted to research and development, the region is marginally better than other underdeveloped areas (Table 7). Employees' skills are the most-expensive and least-elastic resource and an obstacle to technological development in the region. The most-successful efforts to incorporate information and communication technologies in Latin America and the Caribbean have occurred in countries with strong and efficient government and academic institutions committed to invest in education, scientific and technological development, and public services, in tandem with business sectors (for instance, banking and retail commerce) ready and willing to automate their operations.

**Table 7:** The research and educational divide: selected technology inputs by region (1992-1997)*

**Region**	**GNP per capita US$**	**R&D as % of GDP**	**Technicians per 10 ^6^ Population**	**Scientists per 10 ^6^ Population**
OECD Countries	20113	1.8	1326.1	2649.1
Eastern Europe & FSR	4027	0.9	577.2	1841.3
East Asia	6270	0.8	235.8	1026.0
Latin America & Caribbean	5635	0.5	205.4	656.6
Middle East	8941	0.4	177.8	521.0
Sub-Saharan Africa	1971	0.2	76.1	324.3
South Asia	1764	0.8	59.5	161.0

^*^ Adapted from Rodríguez F, Wilson E. From a presentation at a meeting of the InfoDev project of the World Bank; 2000.

### Public Health Authorities Frequently Have a Misguided Vision of ICT

Many public health organizations in Latin America and the Caribbean are not taking advantage of existing ICT opportunities and most existing information systems are inadequate to meeting the requirements of the new models of health care being deployed in the context of health-reform initiatives. Besides the common perception among physicians that health-information systems are mostly a source for scientific and technical information, often public health authorities have a view of clinical-administrative information systems that is obsolete and frozen in a "statistical-epidemiological" archetype, designed for the collection of numerical data representing only counts of events and mostly generating only highly-aggregated statistical data and time series related to mortality, morbidity, and to service utilization and coverage. Those information systems have very little practical interest to direct-care professionals and unit managers and are far behind in providing the logistical and longitudinal individual client-based data required to operate and manage the sort of health care models being deployed in many countries.

Worse still, most public health authorities are totally oblivious to the broad variety of possibilities offered by modern information and communication technologies to manage client-based data, support operations, and mine large databases. Indeed, the health sector has not applied the range of options provided by information and telecommunication technologies as effectively as have other social sectors, and health has been conspicuously underrepresented in national technology-development policies and plans. Such concerns have also been raised by traditional national statistics organizations [27].

As a counterpoint to the passiveness of the public sector, private providers and health groups recognized that a "different" type of information system and data elements are required to run their organizations and survive in a competitive environment driven by increasing consumer demands and expectations and to deliver personalized evidence-based services. Besides using ICT resources to boost productive specialization (such as allowing the efficient use of diagnostic services and consultations, maintenance of integrated records, reduction in the number of specialists, and attainment of economies of scale by linking to national and international markets) there are many new areas of application that are rapidly gaining ground and reducing care costs while improving the continuity and quality of care [28- 30]. The lack of involvement of Latin American and Caribbean public-sector stakeholders in the use of ICT is worrisome. At a time when, in many countries, the ailing, bureaucratic, and inefficient public sector is struggling against poorly-regulated privatization of social services, there is a clear danger in that their inaction in adopting ICT solutions may hasten the further reduction and even the demise of public health services incapable of competing with an information-technology enabled private sector.

### Fast Changing Environments, Untried Business Models, and Excessive Expectations in New Technologies and Processes

Resources, products, and markets that are highly specialized, closed, and regulated are being swiftly opened to new players in a marketplace that is still mostly unregulated and, at the same time, when novel and untried health reform models are being introduced. These circumstances carry with them a very high unpredictability of outcomes. Although businesses and public organizations are adapting with varying speed to new processes and models, the organizational "culture," and the nature and frequency of those business-environment changes may create friction, desirable and undesirable impacts, and personal behaviors that may impede the sequence of the expected project results. Broad objectives are difficult to achieve, the best strategy is to identify the most-repetitive tasks associated with significant costs - eg, the automation of claims and reimbursement procedures - and then proceed area by area. The results of the experience with e-Commerce and e-Business over the last 2 years clearly show that the emergence, adaptation, and real-world deployment of new technologies is a complex issue teeming with uncertainties. For a number of reasons, chiefly related to the technology employed, even in the most-industrialized and computer-literate societies e-Commerce has not developed smoothly.

Unfounded vendor-driven expectations of how the Internet will revolutionize health care have too often overshot their target [31]. Overestimation of results and consequent unfounded expectations is a common pitfall. A common error has been to regard technology as the solution for logistical, administrative, and knowledge-management problems of health care. While, at one extreme one finds technological pessimism and distrust of computer-based solutions, and even some hardcore Luddites driven by the digital-divide concerns, at the other extreme there are truly-excessive and extravagant expectations. The allurement of zero inventory, the oversimplified understanding of business processes, and far-fetched business models with thin or absent margins of return and costly customer acquisition and maintenance strategies have led many e-Companies to bankruptcy. The proliferation of e-Commerce sites of every conceivable nature, clearly not economically viable, resulted in the risk-capital investment bubble that ushered the catastrophic global technology-market collapse of the past year, which also slowed down the deployment of ICT applications in general in the region.

The lesson to be learned for eHealth is that technology is a tool, which can be justified economically only if organizations deploy it in a real-practice environment and closely track how managers and direct-care professionals are using it. This requires the stepwise development and implementation of processes and metrics to monitor productivity and impact [3,31,32].

### Cost Concerns

Web enabling business and government operations is expensive. The United States can be used as a case example: Internet-based marketplaces can lower operational costs and improve efficiencies but deployment expenses will ordinarily cost a typical business US $5.4 million to US $23 million over 5 years. Required procedures involve changing procurement processes, integrating online and internal systems, buying applications, and paying transactions fees and intermediaries. In general, such costs have the following distribution: 32% for internal preparation; 26% for initial contracts and fees; 20% for ongoing internal management; and 22% for ongoing fees and external services [33]. It is difficult for health executives, particularly in the public sector, to justify such levels of investment. There is no data for health ICT expenditures in the region but estimations for all sectors are summarized in Table 8.

**Table 8:** Estimated expenditures in ICT for all sectors in Latin America and the Caribbean in 2001 by category*

**Expenditure Category**	**Value in million US $**	**Percentage**
Hardware	21720	48.9
Personnel	11520	25.9
Software	5760	13.0
Supplies	2220	5.0
Services	1900	4.3
Physical sites	1330	3.0
TOTAL	44450	100

^*^ From Computer Economics, 2001, a fee-based Web service at URL: http://www.computereconomics.com/

### Standardization is a Prerequisite

As providers and insurers soon realized, the simple automation of current processes and services and putting them in a Web-enabled environment is not feasible [3]. A great amount of work has been done in the creation and promotion of data-related standards [12] and, despite the lack of standards in some areas, fortunately there are solutions that allow different organizations and systems to communicate through standardized open-access Internet software languages. Process and data standards for the health care industry involving all constituents - employers, consumers, providers, payers, and regulators - promoted by accrediting organizations have facilitated the adoption of common procedures and routines. A certain amount of standardization also has been driven by regulatory action. In the United States, the introduction of the Health Insurance Portability and Accountability Act (HIPAA) regulations forced a reluctant health industry to adopt uniform formats for health-data exchanges and uniform code sets to identify internal and external health services activities and to be HIPAA-compliant became a requirement of all applications. However, even in developed countries the lack of national standards for unique person identification has slowed implementation of patient-based information systems. An extensive review and reference source on health care data standards was published by the Pan American Health Organization [12].

### Security and Privacy are Major Concerns

Data security and privacy of personal health data are universal concerns and a high-priority issue in many countries. There is a growing concern regarding the protection of health records against intrusion, unauthorized use, data corruption, intentional or unintentional damage, theft, and fraud. Health data transmitted over national and international networks offer unprecedented opportunities for better patient care and community health interventions by facilitating data exchange among professionals but pose new challenges to confidentiality. The promise of the Internet to improve care by timely access to the right information can only be realized through secure connections shared across all platforms.

Given the sensitive nature of health care information, and the high degree of dependence of health professionals on trustworthy records, the issues of reliability (data residing in the electronic health record is accurate and remains accurate), security (owner and users of the electronic health record can control data transmission and storage), and privacy (subject of data can control its use and dissemination) are of particular significance and must be clearly and effectively addressed by health and health-related organizations and professionals. Reliability, security, and privacy are accomplished by the implementation of a number of preventive and protective policies, tools, and actions that address the areas of physical protection, data integrity, access to information resources, and protection against unauthorized disclosure of information [34,35]. A comprehensive review and reference source on personal data protection regulation was published by the Pan American Health Organization [34].

### Quality of Publicly Available Information

This is probably one of the most serious issues in the area of Internet-based interactive health communications. The Internet offers unprecedented power to provide all users of health care information - patients, professionals, families, caregivers, educators, researchers, insurers, regulators, and policymakers - with data of unprecedented timeliness, accuracy, depth, and diversity. Yet it is equally clear that the very qualities that make the Internet such a rich marketplace of ideas - its decentralized structure, its global reach, its leveling of access to the tools of publication, its immediacy of response, and its ability to facilitate free-ranging interchange - also make the Web a channel for potential misinformation, concealed bias, covert self-dealing, and evasion of legitimate regulation. It is very difficult to ascertain and provide recommendations about the credibility, motives, sponsorship, and eventual conflicts of interest in the more than 50000 health Web sites in existence. Many websites are profit driven, others promote unproven and even dangerous forms of treatment or products, while others may be well intentioned, but contain misleading or false information [30,36- 47].

## Recommendations on Policy and Organizational Issues for the Deployment of eHealth in Latin America and the Caribbean

The Presidential Declaration of the 1998 Summit of the Americas and a number of international meetings held since then (Florianópolis Declaration by the representatives of Latin American and Caribbean countries; Brasília Communiqué of the Presidents of South America; Rio de Janeiro Declaration of the Intergovernmental Meeting on ICT for Development; Declaration of the Rio Group; the Declaration of Santiago of the Rio Group; and the European Union Minister's Meeting) made clear that all countries of the Americas have a common stake in improving access to and delivery of health care through communications and information technology. The recommendations presented here follow the spirit of the principles agreed by the governments of the region in the above-mentioned high-level meetings.

### Developing a National Vision, Mission, and Plan of Action for the Public and Private Sectors

The immediate objective is to promote the deployment of core eHealth applications and support functions by incorporating an advanced informatics component into health programs and projects, supported by a combination of funding programs, incentive grant programs, and prototype development funding programs. Elements for the development and implementation of a comprehensive national technology and policy vision, mission, and action plans for eHealth must address the following issues:

- Develop a telecommunications infrastructure that is comprehensive, reliable, ubiquitous, and compatible across applications - such an infrastructure must provide affordable bandwidth that is sufficient to serve the wide variety of users' specific needs. Its development will be dependent upon the continued deregulation of the telecommunications industry and will involve the leveraged use of many ICT technologies that have been spawned by and for other industries.
- Provide technological interfaces that facilitate effective use of the infrastructure and its component systems - these interfaces may involve systems capable of rendering information from multiple modalities, in conjunction with a variety of applications as aids to health services operational support and decision making. They will require modularity and connectivity compliant with standardized interface protocols.
- Implement legal and regulatory infrastructure that will facilitate medical communication - at the professional level, such issues as interstate/province licensure and credentialing of service providers must be addressed, the telecommunications and information industries must develop rational and affordable tariff structures, and legislation must be passed to ensure the protection of personal health information.
- Implement rational and technologically-neutral policies for public and private payers - coverage and payment policies should be established to address the entire range of eHealth applications and technologies. Means should be developed for assessing the appropriateness of health services provided via telemedicine applications. Outcome-based quality-improvement programs will be of great importance in assuring quality and cost-effective medical care.
- Make available appropriate content to consumers, patients, and service providers with the objective of enhancing health care outcomes - the process for conveying quality evidence-based information must permit the user to follow the links between data, inferences, and conclusions. Authentication, access control, confidentiality, integrity, and attribution are key requirements for health-related advice and decision making.
- A retrospective of experiences shows that continuity and sustainability of information-systems projects continue to be a major problem in the region. Externally-funded projects frequently collapse upon funding termination and this demonstrates that all projects need justification in terms of cost-benefit and long-term financial sustainability besides organizational capacity to develop and implement information systems. This further indicates that spreading the financial risk across several stakeholders may be appropriate as cost sharing increases overall awareness, utilization, and long-term potential for success.
- Standards development and implementation must be carried out with the public and private sectors to achieve consensus on a set of information principles for the collection, transfer, storage, and use of health data over national and global information infrastructures. Standards will be defined by a consortium of users; researchers; government, technical and scientific bodies; and the industry at 3 distinct levels: first, in terms of standardization of data and information; second, in terms of the computational facilities required to manipulate and store the information; and third, in terms of telecommunications facilities, employed to transfer information among dispersed sites.

Six areas are envisioned for government involvement in eHealth development and deployment: (a) promotion of education, training, and national planning capacity in information systems and technology; (b) convening groups for the implementation of standards; (c) providing funding for research and development; (d) ensuring the equitable distribution of resources, particularly to places and people considered by private enterprise to provide low opportunities for profit; (e) protecting rights of privacy, intellectual property, and security; and (f) overcoming the jurisdictional barriers to cooperation, particularly when there are conflicting regulations.

The attainment of this mandate involves participation of a large number of stakeholders, but the coordinating effort will necessarily concentrate on the public sector.

### Bridging the Digital Divide

Only a more-active role of government and public-private partnerships in supporting appropriate technology transfer and adaptation through indigenous research and development and the implementation of specific policies to protect local development will create an environment conducive to a reduction of the present ICT development divide. Increasing the general population's capacity to take advantage of information and communication technologies requires heavy investment in general education and training in computer skills. A serious problem for Latin America and the non-English speaking Caribbean is that most of the Internet is directed to native speakers of English and most sites and exchanges are carried out in English. Even physicians, who can generally be expected to have a working knowledge of English, may have problems with such sites. This means that investment is required to develop applications, user interfaces, and contents in national languages.

Developing countries of Latin America and the Caribbean may take advantage of the accumulated knowledge and mistakes and may leapfrog developmental stages; however, this is not expected to be readily achieved due to the barriers posed by the general institutional underdevelopment, low income, illiteracy, and financial constraints that afflict many countries. It is improbable that the bridging of the health-sector development divide will be accomplished easily. In industrialized countries, it took several decades and countless institutional and organizational transformations for the consolidation of economic, institutional, and technological changes and the crystallization of long-term structural patterns necessary for information and communication technologies to spread to vast sectors of the society.

### Developing Organizational and Human Resources: Awareness, Skills, and Leadership

The current health-sector organizational structure and national regulatory framework in Latin America and the Caribbean are not conducive to problem-oriented, interdisciplinary, rapid-response collaborative technical work, and the implementation of political, regulatory, and managerial tasks required to address multifaceted complex technological problems. Organizational and human-resources development through awareness programs, education of health staff, continuous training, and career opportunities must be institutionalized from the inception of the developmental effort. Transference of technical expertise and the appropriation of knowledge by health personnel are necessary for the full participation of end-users in the development process and the best insurance for successful implementations. Success in the deployment of institutional eHealth applications depends on the existence of staff with the right mix of skills in all functions and levels. Recommended strategies include:

- A structured human-resource development program defined with the goal of increasing awareness of eHealth opportunities and training health professionals to assume a leadership role and actively participate in all aspects of systems design and implementation.
- The training strategy will take into account issues associated with: the development, the organizational environment in which systems are expected to operate, and the specific circumstances of the local health system. The following guidelines for training should be implemented: identify target groups based on functions and training needs; develop training programs to meet identified target groups' needs; and establish a network of training focal points, taking into account the specific organization and circumstances of national characteristics and local health-unit requirements and undertakings.
- Target groups to be considered are: those who originate, collect and supply data; operational decision makers (direct health care professionals and administrators); managers, planners, and policy makers; information systems managers; information technology and computing specialists; data analysts; and statisticians and researchers.
- Each country will develop its own strategy for initial and continuing training in health-information systems, considering the overall development of health information systems and its particular health care, educational, research, and market environment.

### Financing and Public-Private Partnerships

Given that the worldwide market for information technology, products, and services in 2001 was valued at US $1086 billion and expected to grow 32% to US $1436 billion by 2004 [48], developing countries need to find ways to share this growing trend. Domestic and foreign, public and private investment sources will be involved, ranging from revenue-sharing initiatives and joint ventures to direct investment, transfer schemes, development funds established by a special tax on telecommunications, major private financial institutions, loans from international funding agencies and development banks, and incentive grants. Joint investment and development involving users, governments, academic and financing institutions and agencies, technical cooperation agencies, and industry interests are seen as necessary. Partnerships with the informatics industry are absolutely fundamental and, in the case of general informatics tools, the industry practically drives the solutions. A concerted effort is needed to secure a clearly-defined and specified partnership with the informatics industry at the global and national levels aimed at application development at acceptable cost. Investments must be attracted to the telecommunications industry by improving investment conditions, lowering duties on telecommunications equipment, and posing no restriction on network design except for technical reasons to allow for new providers.

Development of Health Informatics in the region must be conducted in the context of a framework linking public, private, and social efforts to speed the development of priority ICT solutions. Technical knowledge, experience, and financial investments needed to establish large and complex information system projects require tapping into resources and expertise that no single organization retains. Public and private institutions, academic organizations, the industry, and financing agents must find ways to pool their assets through project partnerships and to add social value to applications of informatics by providing new employment opportunities, socioeconomic development, and educational opportunities, by promoting health, and by supporting cost-effective health services.

### Fostering International Cooperation

In the international setting, cooperation between developed and less-developed countries is essential but special care must be taken to avoid interventionist behavior that ignores users' real needs, fails to understand host capacities, demands action without allowing sufficient time for conceptual assimilation, neglects cultural constraints, and ignores hosts' knowledge basis. As in many other areas of international cooperation the danger is to have too much too soon or too little too late. A possible framework for collaborative work should include support to international health issues, health care reform implementation, application development, education, and economic and technological cooperation.

By demonstrating that social projects, especially health care and education, can be advanced through improved information infrastructure, international technical cooperation and multilateral agencies must collaborate with national and international authorities and experts to demand that funding institutions finance projects in such areas. Consistent with these objectives, governments must demand that international and multilateral agencies must promote and support technical cooperation activities in the development of eHealth, primarily involving knowledge transfer, technical support, facilitation of the exchange of experiences between countries, and fostering the use of appropriate technology and knowledge assets. Priority areas for technical cooperation include: priority assessment, technology evaluation and selection criteria, implementation issues, emerging technologies linking patients and providers, access to knowledge databases, consumer informatics, and the utilization of Internet and Internet-enabled technologies.

### Creating Incentives Through Regulation

Many Latin American and Caribbean countries are committed to reform their telecommunications systems. They recognize that progress in the telecommunications sector is essential to establishing health informatics and to ensuring the global competitiveness of their economies, and attention has been on liberalizing the markets. Recommendations include actions in the following areas: market access issues (interconnection-regulation framework, clear and transparent regulation governing competition, and allocation of spectrum harmonization [ie, assignment of the same frequency bands everywhere]); standards (interoperability standards and the streamlining and liberalization of the conformity-assessment process for equipment certification); regulation (elimination of rules of origin and treating products from different countries equally when standards are the same, the elimination of subsidies, antidumping practices, and abolishment of countervailing duties); promoting competition (establish a regulatory framework that balances national needs in the context of creating a competitive national telecommunications system, weigh cost of delaying competition against the need for an effective transitional regime, and move towards full liberalization as quickly as appropriate); and protecting technology and intellectual-property rights.

Experts agree that to be effective and efficient the health care industry must operate in a digital environment, encompassing connectivity, commerce, and community/content sites. But no one can pinpoint exactly when everything will converge. Nudging the health sector toward compliance is a valid and effective approach. The European and Canadian health care systems have used this strategy and HIPAA, the US federal Health Insurance Portability & Accountability Act, is a prime example of how the industry can be coached into complying with a variety of guidelines related to standardization, security, and privacy. In effect, HIPAA is forcing an eHealth solution on the US health care industry.

## Conclusions

Health care organizations in Latin America and the Caribbean, particularly in the public sector, are not yet prepared to adopt ICT effectively. The goal of a national health ICT vision and strategic plan of action is to establish a coherent national arrangement directed to facilitating projects and infrastructure development, maximizing the benefits for invested financial resources, and enabling people to accept and function more effectively in an informatized, evidence-based, and competitive health practice environment.

Governments should focus on their role as sponsors of basic scientific and technological research, bridging the digital divide, fostering public-private partnerships, managing international cooperation efforts, and establishing the regulatory and incentive components. All stakeholders must work collaboratively to grapple with the many standardization and infrastructure-development issues and the transnational and global eHealth aspects that must be addressed in a comprehensive manner. International aspects of eHealth services form a critical and urgent area still to be addressed by the World Trade Organization and regional trade blocks. Legislation proposals should be initiated to ensure that the technology does not abridge patients' rights to confidentiality or security of medical records, and that agreement on practice parameters be developed to include aspects related to informed consent, physician liability, nonphysician liability, reimbursement, practice standards, and physician-patient relationships.

The public sector, the industry, and partnerships have the responsibility for assuming an active leadership role in educating the medical community and in coordinating and encouraging the effective implementation of relevant applications. Health organizations must be provided with information about the opportunities as well as the risks of eHealth solutions. Technology-evaluation sources and results must be made available and health managers must be guided in the difficult process of specifying systems, procuring, acquiring, and contracting for ICT products and services. Knowledge repositories must be established in cooperation with the industry, centers for technology evaluation, academic research groups, and centers of excellence.

## Abbreviations

GDP: Gross Domestic Product
GNP: Gross National Product
HIPAA: Health Insurance Portability and AccountabilityAct
ICT: Information and Communication Technologies
PPP: Purchase Power Parity
